# Slip-knot thread method for performing anchor-wire technique to prevent proximal dislocation of preceding stent in placement of multiple biliary inside plastic stents

**DOI:** 10.1055/a-2512-5264

**Published:** 2025-01-23

**Authors:** Yuichi Hirata, Mariko Hatada, Kana Miyara, Yuichiro Aoyama, Ryosuke Mizukami, Daisuke Orita, Yoshihiro Okabe

**Affiliations:** 1 469536 Gastroenterology, Kakogawa Central City Hospital, Kakogawa, Japan

Endoscopic biliary drainage using “inside plastic stents” (iPSs) is favored for patients with unresectable malignant hilar obstruction, because it offers prolonged stent patency and easy reintervention 1 . However, one of the problems encountered in the endoscopic placement of multiple iPSs is proximal dislocation of the preceding iPS during the subsequent iPS insertions. Although several methods for preventing preceding iPS migration have been reported 2 3 , some devices are not immediately available. Herein, we report a case in which a slip-knot was created using the threads of the preceding iPS ( Fig. 1 ), to to perform the anchor-wire technique 4 for preventing its proximal dislocation during the endoscopic placement of the subsequent iPS.

**Fig. 1:**
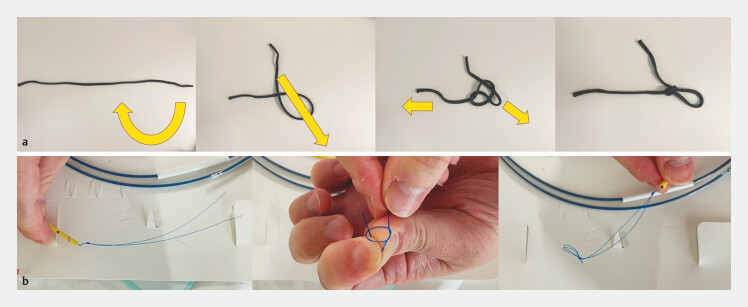
**a** A slip-knot, formed as shown here, can be untied by simply pulling one end. **b** Slip-knot created using the threads of an inside plastic stent (iPS).

A 72-year-old man with malignant hilar obstruction due to gallbladder cancer was admitted to our institution for acute cholangitis associated with recurrent biliary obstruction ( Fig. 2 ). Endoscopic retrograde cholangiopancreatography was performed, and the initial iPS (Through & Pass IS; Gadelius Medical, Tokyo, Japan) was removed. Although bilateral drainage using iPSs was necessary, proximal dislocation of the preceding iPS was anticipated because the biliary stenosis was severe. Therefore, a slip-knot was created in the threads of the first iPS, before it was inserted. Because the preceding iPS was almost dislocated proximally during the subsequent iPS insertion, anchor-wire technique was performed using the thread’s loop formed with slip-knot 4 . Following this, the subsequent iPS was successfully inserted into the intrahepatic bile duct through the hilar obstruction without dislocation of the preceding iPS and placed in the appropriate position. After placement of the iPSs, the slip-knot threads were straightened by pulling the end with a biopsy forceps passed through the working channel of the duodenoscope ( Fig. 3 , Video 1 ). This procedure requires only commonly used biopsy forceps additionally and is easy to do.

**Fig. 2:**
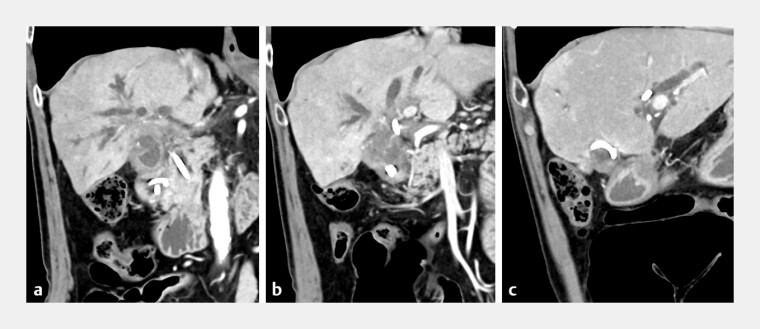
Computed tomography images. A 72-year-old man with malignant hilar obstruction due to gallbladder cancer was admitted to our institution for acute cholangitis associated with recurrent biliary obstruction.

**Fig. 3:**
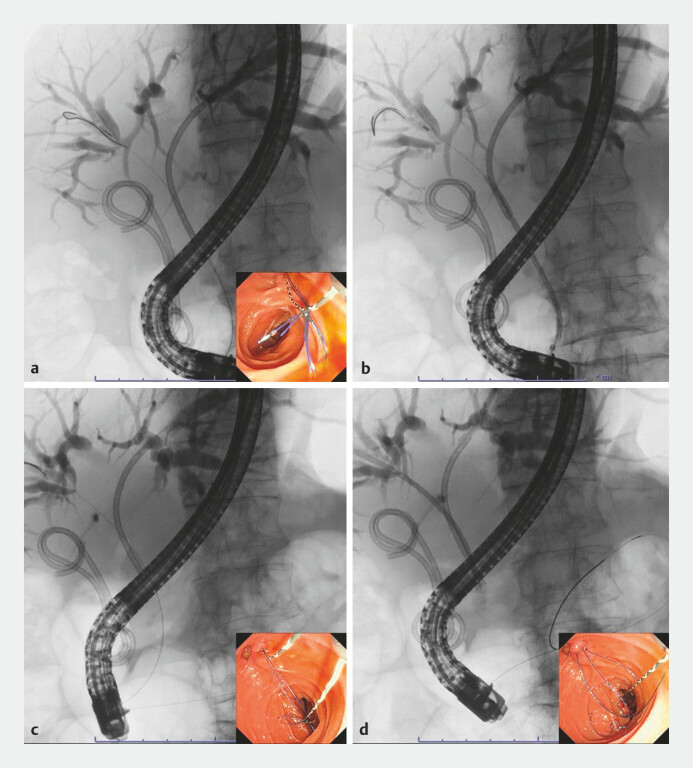
Fluoroscopic images of the procedure using the slip-knot method. **a** The first inside plastic stent (iPS), with a slip-knot in its threads, was inserted. **b** This preceding iPS was almost dislocated proximally during the subsequent iPS insertion. **c** Therefore the anchor-wire technique was performed using the slip-knot loop in the threads of the preceding iPS. **d** The subsequent iPS was successfully inserted into the intrahepatic bile duct through the hilar obstruction, without dislocation of the preceding iPS, and placed in the appropriate position.

The slip-knot thread method for performing the anchor-wire technique to prevent proximal dislocation of the preceding stent during placement of multiple biliary inside plastic stents (iPSs).Video 1

The slip-knot thread method for performing the anchor-wire technique is useful for preventing proximal dislocation of the preceding iPS during the endoscopic placement of multiple iPSs.

Endoscopy_UCTN_Code_TTT_1AR_2AZ

## Notes

Endoscopy E-Videos https://eref.thieme.de/e-videos
*E-Videos* is an open access online section of the journal *Endoscopy* , reporting on interesting cases and new techniques in gastroenterological endoscopy. All papers include a high-quality video and are published with a Creative Commons CC-BY license. Endoscopy E-Videos qualify for HINARI discounts and waivers and eligibility is automatically checked during the submission process. We grant 100% waivers to articles whose corresponding authors are based in Group A countries and 50% waivers to those who are based in Group B countries as classified by Research4Life (see: https://www.research4life.org/access/eligibility/ ). This section has its own submission website at https://mc.manuscriptcentral.com/e-videos .
